# Draft Genome Sequence of the Chronic, Nonclonal Cystic Fibrosis Isolate *Pseudomonas aeruginosa* Strain 18A

**DOI:** 10.1128/genomeA.00001-13

**Published:** 2013-03-21

**Authors:** Jerry K. K. Woo, Kerensa McElroy, Scott A. Rice, Sylvia M. Kirov, Torsten Thomas, Staffan Kjelleberg

**Affiliations:** a School of Biotechnology and Biomolecular Sciences, Centre for Marine Bio-Innovation, University of New South Wales, Sydney, Australia; b The Singapore Centre on Environmental Life Sciences Engineering, Nanyang Technological University, Singapore; c School of Medicine, University of Tasmania, Tasmania, Australia

## Abstract

*Pseudomonas aeruginosa* strain 18A is a clinical, nonclonal isolate retrieved from the sputum of a chronically infected cystic fibrosis patient. The genome of 18A was sequenced for comparison with environmental and clinical isolates to identify genes that might facilitate its persistence during infection.

## GENOME ANNOUNCEMENT

*Pseudomonas aeruginosa* is a major factor in the mortality of cystic fibrosis (CF) patients. Although several studies have adopted a genomic approach to identify potential virulence determinants of laboratory type strains (1, 2) and clonal CF *P. aeruginosa* isolates (3, 4), there are limited studies on nonclonal isolates (3). We have sequenced the nonclonal, chronic *P. aeruginosa* isolate 18A.

A combination of the 454 FLX titanium system (Roche) and the paired-end Illumina genome analyzer GAII (Illumina) sequencing was used. We performed a hybrid assembly using all of the 454 reads and a subset of the Illumina reads in MIRA3 and obtained average coverage of 59× for 454 reads and 147× for Illumina reads for contigs with length greater than 500 bp. MIRA3 flags were set to perform accurate *de novo* genome assembly, utilizing paired-end information for the Illumina data and XML trace information associated with the 454 FLX data. All resulting contigs with lengths greater than 500 bp were manually finished in Gap5, with contigs being merged if their ends overlapped and were not flagged as repeat sequences by MIRA3. After merging, all remaining contigs ended either in repeats or in areas of no coverage. Finally, we tested misassembly by aligning all Illumina paired-end reads against the assembled contigs using Novoalign V2.07.06 and screening for pairs with unusual fragment lengths or orientation. The final assembly consisted of 179 contigs. It was annotated on the RAST (Rapid Alignment using Subsystems Technology) (5) server, and Glimmer (6) was used to identify open reading frames (ORFs).

The draft genome of *P. aeruginosa* 18A yielded a total of 6,093,587 bp, with a 66.4% GC content, consistent with the GC content (66.2% to 66.6%) for *P. aeruginosa* strains (1–3, 7). A total of 5,453 ORFs were identified in strain 18A, of which 96.6% (5,267 of the ORFs) were common to the genome of strain PAO1. The unfinished 18A genome lacked 303 genes found in PAO1, including metabolic genes for purine and pyrimidine (*rbs* operon), mannitol (*mtl* operon) (2), branched-chain amino acid (*bkd-lpdV* operon), gluconate (8, 9), and production of the Psl exopolysaccharide (*psl* operon) (10). Ten genomic islands were present in 18A that were absent in PAO1, including a flagellar glycosylation island (11), a *P. aeruginosa* genome island 1 (PAGI-1) (12), and a cluster of regularly interspaced short palindromic repeats (CRISPR) (13). The 18A genome also contained genes that were absent from the PAO1 genome but have been identified in clonal clinical isolates such as PA14 (1) and LESB58 (4). These include the pyoluteorin biosynthesis locus and the O-antigen biosynthesis locus. The 18A genome was found to have type I pilin genes, in contrast to the type II and type III genes of strains PAO1 and PA14, respectively. While 18A shares a significant amount of genome content with strain PAO1, it appears to also have a mosaic of genes from clonal clinical isolates and environmental isolates, which may be associated with its nonclonal distribution.

### Nucleotide sequence accession numbers. 

The draft genome and annotation are accessible from the EMBL database under accession numbers CAQZ01000001 through CAQZ01000179.
